# Early Season Symptoms on Stem, Inflorescences and Flowers of Grapevine Associated with *Botryosphaeriaceae* Species

**DOI:** 10.3390/plants9111427

**Published:** 2020-10-24

**Authors:** Pedro Reis, Ana Gaspar, Artur Alves, Florence Fontaine, Inês Lourenço, José Saramago, Mariana Mota, Cecília Rego

**Affiliations:** 1 LEAF—Linking Landscape, Environment, Agriculture and Food, School of Agriculture, University of Lisbon, 1349-017 Lisbon, Portugal; anapatriciagaspar@hotmail.com (A.G.); mariana@isa.utl.pt (M.M.); crego@isa.ulisboa.pt (C.R.); 2 CESAM—Centre for Environmental and Marine Studies, Department of Biology, University of Aveiro, 3810-193 Aveiro, Portugal; artur.alves@ua.pt; 3 SFR Condorcet FR CNRS 3417, Université de Reims Champagne-Ardenne, Résistance Induite et Bioprotection des Plantes EA 4707, BP 1039, 51687 Reims Cedex 2, France; florence.fontaine@univ-reims.fr; 4 BASF Portuguesa, S.A. Rua 25 de Abril, 1, 2689-538 Prior Velho, Portugal; ines.lourenco@basf.com (I.L.); jose.saramago@basf.com (J.S.)

**Keywords:** grapevine, *Diplodia seriata*, *Neofusicoccum parvum*, pathogenicity, diagnostic

## Abstract

Botryosphaeria dieback caused by several *Botryosphaeriaceae* species is one of the most important grapevine trunk diseases affecting vineyards worldwide. These fungi cause wedge-shaped perennial cankers and black streaking of the wood and have also been associated with intervein leaf chlorosis, dried or mummified berries, and eventually, the death of the plant. Early season symptoms may sometimes be disregarded by growers, being mistaken with symptoms from other diseases such as downy mildew or botrytis rot. Currently, few studies are available to determine what species may be causing these early season symptoms in grapevines. During the 2018 season, during the flowering period, grapevine samples showing necrosis on green shoots, dried inflorescences, and flowers, were collected in vineyards throughout the central regions of Portugal. Isolations were performed from symptomatic organs, and twenty-three isolates of *Botryosphaeriaceae* were selected. An analysis of the ITS and part of the translation elongation factor 1-α sequences was performed, revealing that the two main species apparently responsible for these symptoms were *Diplodia seriata* and *Neofusicoccum parvum*. In pathogenicity tests conducted on 1-year-old plants grown under controlled conditions in a greenhouse and on field-grown clusters, symptoms were reproduced, confirming the pathogenic behavior of the selection of isolates.

## 1. Introduction

Grapevine trunk diseases (GTDs) are one of the most critical problems affecting viticulture worldwide, causing yield reduction and increased production costs adding to the existing cost of the annual vineyard operations. In fact, these diseases pose a significant threat to sustainable viticulture worldwide since the cost associated with GTD losses has been increasing during recent years. For example, in Spain, there was an increase of 1.8% of incidence in vineyards in 2003 up to 10.5% in 2007 [1]. In France, it is estimated that the equivalent of 1 billion euros is lost every year due to these diseases [2], while in Australia, the economic impact can reach up to 8.3 billion AUD [3]. GTDs include three main fungal diseases—eutypa dieback, esca disease and botryosphaeria dieback—that involve one or several species of fungi [4,5,6]. In Portugal, botryosphaeria dieback and Esca are considered the major diseases affecting adult grapevines, causing considerable economic losses, in all the country’s vine growing regions [2].

Botryosphaeria dieback is, currently, one of the most important GTDs worldwide [4], caused by fungi in the *Botryosphaeriaceae* family. Fungi belonging to this family have been found worldwide as endophytes, saprophytes or pathogenic in many perennial and ornamental plants [7,8,9,10].

Twenty-six species in different *Botryosphaeriaceae genera* have been associated with botryosphaeria dieback in grapevines [11,12,13,14,15,16,17]. The most common species isolated from grapevines are *Diplodia seriata* [18,19,20,21,22,23,24], *Diplodia mutila* [25,26], *Neofusicoccum parvum* [27], and *Lasiodiplodia theobromae* [16,28,29,30].

In different countries, species occurring on grapevines have been shown to differ in pathogenicity, in their epidemiology, and in the symptoms produced [31]. Therefore, the symptomatology of botryosphaeria dieback is somewhat complex. However, the most common symptoms include no or limited burst, perennial cankers, trunk dieback, wood necrosis, vascular streaking, and plant death [16,28,31,32,33,34]. Infection of grapevines by *Botryosphaeriaceae* can also lead to leaf and berry symptoms, where yellowish-orange or wine-red spots on leaf margins and blades can appear, depending on the cultivar (white or red) [4,20,35,36,37].

During the 2018 season, growers and field technicians in the central region of Portugal, started to observe symptoms characterized by necrosis on the base of green shoots, which could lead to the complete detachment of the shoot later in the season, wilting of the apex of the shoot, wilting of leaves, and more important, necrosis on the peduncle and rachis of the developing clusters, drying of inflorescences and flowers. These symptoms appeared even after the planned fungicide applications against other grapevine diseases, which was puzzling for the growers. Samples were sent for analysis in our laboratory and revealed the presence of *Botryosphaeriaceae* on the tissues analyzed. Although the epidemiology and distribution of *Botryosphaeria* canker in the wood of grapevines as well as other hosts have been widely studied, the relationship between *Botryosphaeriaceae* and these symptoms have not yet been investigated and no studies were carried out to understand which species may be causing these early season symptoms. The objective of this study was thus to identify the pathogens of these early season symptoms in vineyards of Portuguese central regions by means of morphological features, DNA analysis, and pathogenicity tests.

## 2. Results

### 2.1. Sampling and Fungal Isolation

A total of 42 *Botryosphaeriaceae* were isolated from the samples received from the 17 vineyards, and from several different symptomatic organs, including apex and base of the shoots, clusters, and leaves. Other fungi isolated from these samples were *Alternaria alternata*, *Phomopsis* spp. and *Penicillium* spp. The majority of *Botryosphaeriaceae* isolates were obtained from the symptomatic tissue collected at the apex of the shoot (36%), followed by the base of the shoots (33%). Isolates obtained from the rachis of the clusters represented 17% while 9% were from the clusters (flowers and inflorescences) and only 5% from the leaves. From this set of isolates, a total of twenty-three isolates (Table 1) were selected based on their cultural characteristics, with the attention of keeping at least one isolated obtained from each vineyard. These isolates were characterized and identified based on morpho-cultural characters, DNA sequencing, and pathogenicity tests.

### 2.2. Morphological Characterization and DNA Analysis for Fungal Identification

Isolates of *Botryosphaeriaceae* could be split into six groups based on colony morphology, after 8 days of growth on PDA. After promoting sporulation of the isolates under study on 2% water agar medium with autoclaved pine needles, three groups were distinguished based on the morphology of conidia. One group containing 12 isolates produced conidia initially hyaline, becoming dark brown, aseptate and ovoid, with average dimensions of (20.95–) 23.60 ± 1.15 (–26.46) × (7.79–) 9.18 ± 0.5 (10.61), which were determined to be *D. seriata* based on conidia morphology and DNA analysis (Table 2). A second group with 3 isolates produced ellipsoidal conidia, with flat base, unicellular and hyaline, which could develop 1 to 2 septa over time, with average dimensions of (14.18–) 16.39 ± 1.95 (–19.46) × (4.50–) 5.19 ± 0.13 (–6.02) which were determined to be *N. parvum*, based on conidial morphology and DNA analysis (Table 2). Finally, there was one isolate that formed hyaline and aseptate conidia, oblong to ovoid with both ends broadly rounded and with dimensions of (20.80–) 23.50 ± 1.41 (–25.05), which was determined to be *D. mutila* based on above-mentioned parameters (Table 2). There were seven isolates that did not sporulate until the end of this work, but their species identification was determined through DNA analysis.

### 2.3. Pathogenicity Tests

#### 2.3.1. Pathogenicity Tests on Tendrils and Leaves

**Figure 1:**
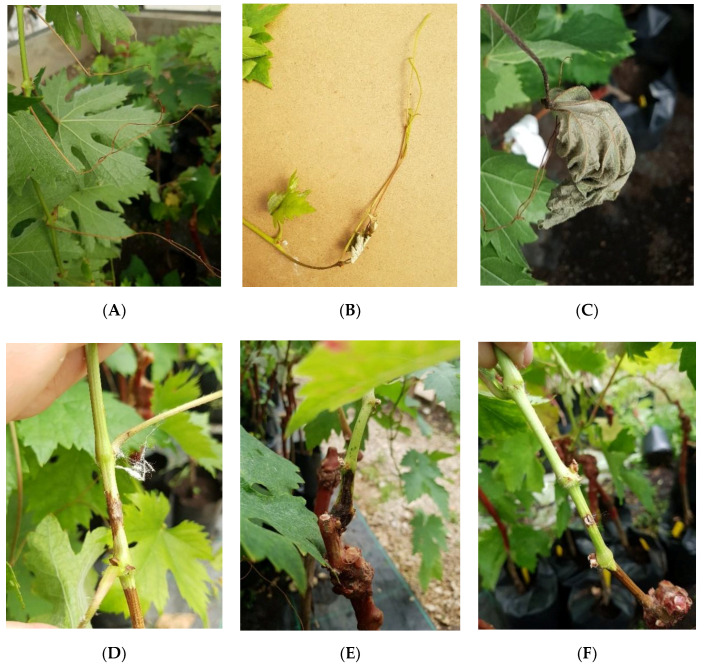
Symptoms observed on tendril and green shoots: (**A**) necrotic tendrils obtained after inoculation with *D. mutila* (Bt218); (**B**) necrotic apex of the shoot after inoculation with *D. seriata* (Bt204); (**C**) necrotic leaf after inoculation with *N. parvum* (Bt216); (**D**) lesion obtained after inoculation with *D. seriata* (Bt204); (**E**) lesion obtained after inoculation with *N. parvum* (Bt216); (**F**) control inoculation.

**Figure 2:**
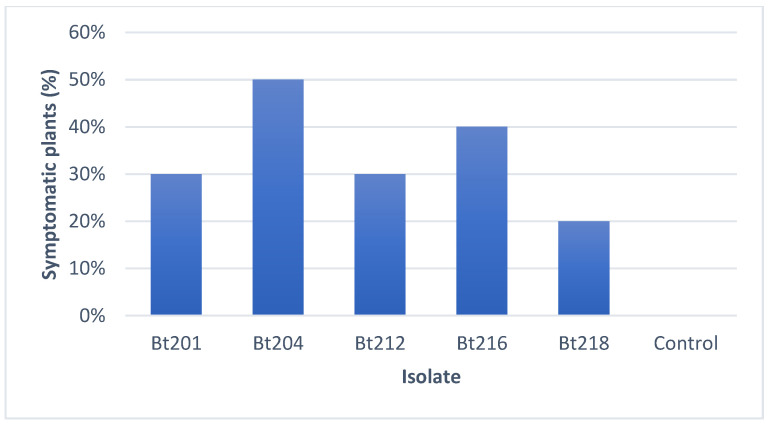
Percentage of plants showing symptoms on tendrils and leaves, after inoculation with the *Botryosphaeriaceae* isolates under study. Ten plants were used per isolate while control plants were sprayed with sterile distilled water.

All the isolates tested were pathogenic towards the Aragonez 1-year-old grafted cuttings used in the greenhouse experiment, and they were able to, in some extent, reproduce symptoms closely resembling the ones observed on the field survey, namely necrotic tendrils, apex of the shoots and leaves (Figure 1A–C). Isolate Bt204 identified as *D. seriata*, was the isolate where the highest percentage (50%) of plants showed the above-described symptoms, followed by isolate Bt216 identified as *N. parvum* (40%) (Figure 2). The isolate with the lowest percentage of infected plants (20%) was Bt218 which was identified as *D. mutila*. No control plants showed any symptoms of disease, growing normally during the experimental period.

#### 2.3.2. Pathogenicity Tests on Green Stems

**Figure 3:**
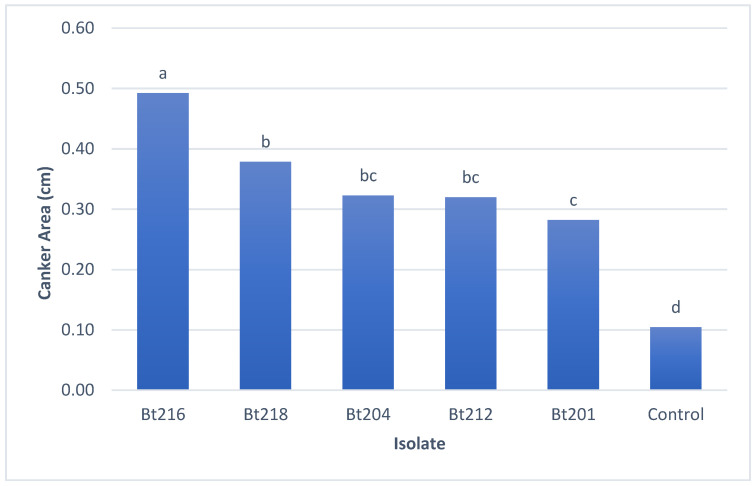
Mean canker areas (cm) in green stem caused by artificial inoculation with *Botryosphaeriaceae* isolates under study. Different letters in column correspond to significant differences (*p* < 0.05) based on ranks assessed by Kruskal-Wallis analysis. Ten plants were used per isolate while control plants were sprayed with sterile distilled water.

All isolates under study proved to be pathogenic towards 1-year-old grafted Aragonez grapevines, by being able to produce lesions in the inoculated tissues (Figure 1D–F). All the isolates showed significant statistical differences when compared to the control plants (Figure 3), being the largest average lesions recorded for isolate Bt216 (*N. parvum*) which showed significant differences towards the remaining isolates under study. The smallest average lesions were recorded for three isolates identified as *D. seriata* (Bt201, Bt204 and Bt212), while isolate Bt218 (*D. mutila*) showed lesions on average between those produced by *N. parvum* and *D. seriata* (Figure 3)

#### 2.3.3. Field Pathogenicity Tests on Clusters

**Figure 4:**
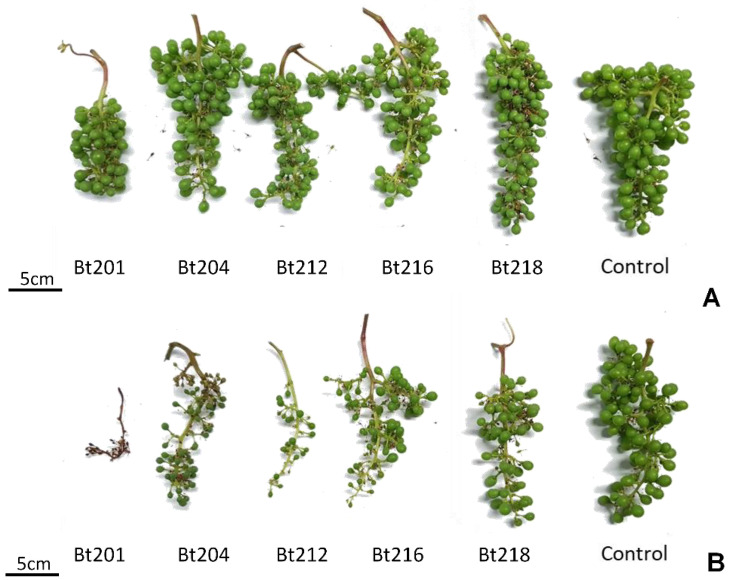
(**A**) best cluster from each inoculation/isolate; (**B**) worst cluster from each inoculation/isolate.

**Figure 5:**
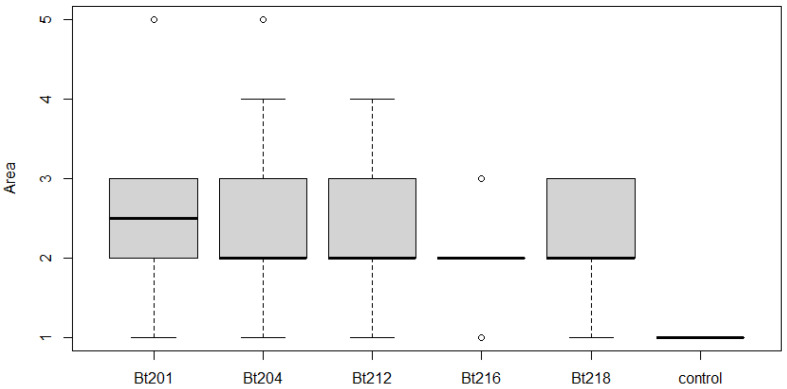
Boxplot of the percentage of infected area on clusters artificially inoculated with *Botryosphaeriaceae* isolates under study. Scale of evaluation of area infected—1 = no symptoms; 2 = 1–5%; 3 = 5–25%; 4 = 25–50%; 5 = >50%. The median is represented by the solid line. Top and bottom lines of the box correspond to the 25th and 75th percentiles of the data, respectively. Circles represent outliers.

All isolates used in this study were able to cause symptoms of dried berries and inflorescences on inoculated clusters (phenological stage EL 25 to 27) of Cabernet Sauvignon (Figure 4A,B). Although only an exploratory statistical analysis was possible based on the infection scale established, due to the low number of repetitions (*n*), all isolates showed a significant different proportion of symptomless (1-ranked) samples (*p* < 0.05) towards the control, therefore confirming the pathogenicity of the isolates under study when artificially infecting field-grown clusters. Lower *p*-values (*p* = 0.0049) for isolates Bt201 and Bt218 may suggest a stronger ability of these two isolates in causing symptoms on clusters when comparing to the remaining isolates which recorded a higher p value (*p* = 0.0182). In order to address more in detail this topic, a boxplot analysis was performed (Figure 5) and results showed that among the different isolates, Bt216 showed the lowest average infected area, as nearly all the samples were ranked as a 2-class, and no sample ranked more than 3-class, corresponding to less than 25% of affected area. For the isolates Bt212, Bt218 and Bt204, half of the samples, showed more that 25% of the cluster area with infection symptoms. Within these three isolates, Bt218 seems to be less aggressive, as no samples were quoted as 3-class or more severe. Bt201 suggested once more to showed tendentially higher infected area values.

For all the pathogenicity experiments, pathogens were recovered from symptomatic tissues of all infected plants, while no *Botryosphaeriaceae* isolates were re-isolated from control plants.

## 3. Discussion

This is the first study aimed at describing grapevine early season symptoms associated with Botryosphaeriaceous fungi in Portugal, including necrosis and wilting of the apex of the green shoots, wilting of leaves, necrosis on the peduncle and rachis of the developing clusters and, drying of inflorescences and flowers. Morphological studies and DNA sequence analysis allowed to identify the presence of three different species of *Botryosphaeriaceae*: *D. seriata*, *N. parvum* and *D. mutila*. The most common species found in our study causing the described symptoms was *D. seriata*, since 74% of the isolates belonged to this species, which agrees with previous studies such as Auger et al. [40] in Chile, Luque et al. [29] in Spain and Carlucci et al. [41] in Italy, this being one of the most common species associated with botryosphaeria dieback in vineyards. All the species identified in the present study have been previously identified in other grape-growing regions worldwide, and they have been linked with a broad range of symptoms, including leaf spots, fruit rot, shoot dieback, bud necrosis, vascular discoloration of the wood and perennial cankers [16]. All isolates tested showed the ability to cause symptoms to some extent, similar to those observed in the field. Regarding the ability for causing necrosis on tendrils and leaves, all the isolates showed few differences on the percentage of infected plants, being the only exception, *D. mutila* which was able to only cause symptoms on 20% of the plants. Considering the potential of causing lesions on green stems, the isolate belonging to the species *N. parvum* (Bt216) was able to induce the largest average lesions, whereas the lowest average lesions were recorded for the isolates belonging to *D. seriata*. These results are in accordance with previously described results regarding aggressiveness of *Botryosphaeriaceae* species [16], in which *N. parvum* is considered to be highly aggressive towards grapevine, while *D. seriata* is considered to be only mildly aggressive. In this case, *D. mutila* showed intermediate average lesions values contrary to what was observed on the pathogenicity tests on both tendrils and leaves, where this isolate appeared to be the least aggressive. More epidemiology studies should be performed on the behavior of these species to understand if there is a difference of aggressiveness towards different types of grapevine tissues, or if the differences observed were due to experimental design. Considering the ability to cause symptoms on clusters, such as dried berries and inflorescences, the highest average infected area was recorded for *D. seriata* and *D. mutila*. These results are contrary to the ones obtained for the other pathogenicity tests. This may be not due to the aggressiveness of the isolates/species, but to the differences on experimental conditions. These pathogenicity tests were conducted on field-grown grapevines while the other tests were conducted on grapevines growing in a greenhouse-controlled environment, not to mention the differences in plant age.

Although the main economic impact fungi of the *Botryosphaeriaceae* family are associated with the trunk and cane symptoms, damage by these fungi showing up so early in the growing season should not be overlooked, since they could perform an important role in their epidemiology and become a source of inoculum for wound infections leading to trunk diseases. *Botryosphaeriaceae* fungi occur in most parts of the world and are found as endophytes or parasites and saprophytes on a vast number of both annual and perennial plants [16]. Infection by *Botryosphaeriaceae* is considered to occur mainly through pruning wounds [16,42,43], since cankers start to develop from wounds on leaves, branches or stems. However, several studies have shown that these fungi can infect through lenticels, stomata, or other openings on healthy plants [44,45,46,47,48]. Nowadays, pruning wounds are considered as the main door of infection for *Botryosphaeriaceae* on grapevine. Nonetheless, Shafi et al. [48] recently showed by fluorescence microscopy that these pathogens can remain latent on the grapevine bark, and even without any type of wounding, germinating conidia and mycelium could be observed near lenticels, as well as mycelia in the underlying wood, demonstrating that the pathogens had entered through the lenticels. Therefore, our observation suggests that *Botryosphaeriaceae* fungi may thus have the ability to colonize and infect healthy grapevine tissues, even without wounding, which may be one of the reasons for the symptoms observed on these vineyards during the spring of 2018.

Pycnidia of *Botryosphaeriaceae* associated with dieback disease can be detected in old pruning wounds, infected spurs, embedded in the bark of the cordons or trunk of infected grapevines, and also on pruning debris left in the vineyard [16,49,50,51]. In France, Kuntzmann et al. [52] reported that conidia of *Botryosphaeriaceae* were released during the whole vegetative period, but *D. mutila* released its spores later that *D. seriata*, indicating that either these fungi differ in their ability to grow and sporulate or that they merely differ in their response to meteorological conditions. These authors also reported that in their study 50% of the spores of *D. mutila* were captured during late summer and autumn while most of the conidia of *D. seriata* were captured during the spring months. This is in accordance to our findings, especially when taking into consideration that our sampling took place between the months of March and July, since most of the species found during our study, belonged to *D. seriata*, while only one isolate of *D. mutila* was recorded. As stated before, our study was conducted when grapevines were at the E-L 23–25 phenological stage which corresponds exactly to the flowering period which has been reported previously by Spagnolo et al. [53] to be the most sensitive period to infection by botryosphaeria dieback agents, as a consequence of the high metabolic activity leaning towards the development of flowers. Therefore, we believe that the conjunction of all these factors, presence of inoculum due to precipitation during the spring months, associated with the phenological stage of the grapevines and the ability of these fungi in infecting through other pathways other that wounds, may have a strong influence on the manifestation of early season symptoms caused by *Botryosphaeriaceae*. Thus, planning further research is strongly advised on this subject with special attention for epidemiology and pathogenicity studies to determine the infection pathway and infection moments regarding environmental conditions.

To the best of our knowledge, this is the first study aimed at describing these *Botryosphaeriaceae* symptoms in grapevines. In conclusion, our work has demonstrated that Botryosphaeria dieback fungi, mostly associated with wood cankers, appear to have the potential to cause serious early season symptoms, since the pathogenicity tests conducted with all the species found were able to reproduce these symptoms on several different grapevine organs. Nevertheless, we strongly believe that further research is needed on this subject by collecting more samples from different regions of Portugal and by testing a wider range of isolates, to try to understand what are the most common species involved in the expression of these symptoms, and what is or if there is an influence of geographical location and climate conditions [54,55]. The knowledge and clarification of the symptoms caused by these fungi and the development of proper diagnostic may help growers not to confuse them with symptoms of other diseases, and to set up a proper management plan.

## 4. Materials and Methods

### 4.1. Sampling and Fungal Isolation

During the early spring of the 2018 season, at the phenological stages E-L 23–25 [56], samples from 17 vineyards spread throughout the central regions of Portugal, namely Lisboa, Tejo, and Alentejo, were received at Instituto Superior de Agronomia (ISA). These samples showed necrosis on shoots and dried inflorescences and flowers, and isolations were made by cutting several pieces from symptomatic organs. Pieces collected were surface disinfected with a 7% sodium hypochlorite solution, rinsed in sterile distilled water (SDW) and plated onto 9 mm Petri dishes containing Potato Dextrose Agar (PDA, BD Difco, Sparks, MD, USA) amended with chloramphenicol (PanReac AppliChem, Darmstadt, Germany) at 250 mg/L. After incubation at 25 °C for one week, Petri dishes were assessed for the presence of *Botryosphaeriaceae* colonies which were sub-cultured onto fresh PDA dishes and, again incubated at 25 °C for one week, in darkness. All isolates obtained were stored in the collection of the ISA, Lisbon, Portugal, and were afterwards characterized morphologically, as well as properly identified by DNA sequence analyses.

### 4.2. Morphological Characterization and DNA Analysis for Fungal Identification

#### 4.2.1. Morphological Characterization

Isolates under study were, plated onto 6 mm Petri dishes containing 2% water agar with autoclaved pine needles (*Pinus pinea*) and incubated at 25 °C under fluorescent light, in order to promote sporulation [27,28,34]. Pycnidia were mounted on microscope slides in a solution of lactophenol blue, and digital images were recorded with a Leica DFC295 camera on a Leica DM 2500 microscope at a 400× amplification. Twenty conidia were measured with the Leica Suite v3.16 program, for each isolate under study. Dimensions of the conidia are given as the range of dimensions with minimum and maximum dimensions in parentheses followed by mean and standard deviation.

#### 4.2.2. DNA Analysis

DNeasyTM Plant Mini Kit by Qiagen^®^ (Venlo, The Netherlands) was used to extract genomic DNA from 8-day-old cultures grown in PDA and incubated at 25 °C, in the darkness. The ITS region was amplified using primers ITS5 and ITS4 [57], while the primers EF1-688F and EF1–1251R [58] were used to amplify part of the elongation factor 1α gene. The PCR mixtures contained 1 × PCR buffer REDTaq Ready Mix (Sigma-Aldrich, Saint Louis, MO, USA), 3 mM MgCl_2_, 0.4 mM dNTP mix, 12.5 pmol of each primer, 0.06 unit/μL of Taq Polymerase and 25–50 ng of template DNA. Each reaction volume was made up to 25 μL with sterile ultrapure water. Negative controls with sterile ultrapure water instead of the template DNA were used in every reaction. The amplification conditions for ITS were as follows: initial denaturation of 5 min at 95 °C, followed by 40 cycles of 30 s at 94 °C, 30 s at 58 °C, 1 min and 40 s at 72 °C and a final extension period of 10 min at 72 °C. For the amplification of part of the tef1-α gene, the conditions were, an initial denaturation of 2 min at 94 °C, followed by 35 cycles of 30 s at 94 °C, 45 s at 55 °C, 1 min at 72 °C and a final extension period of 10 min at 72 °C. Each amplicon was separated by electrophoresis at 120 V for 30 min in a 1% agarose gel in 0.5 × TBE buffer. Gels were stained with 3 μL of GreenSafe Premium (Nzytech, Lisbon, Portugal), and were visualized using with a UV transilluminator to assess PCR amplification.

The amplified PCR fragments were purified using an Illustra ExoProStar Enzymatic PCR and Sequencing Clean-up Kit (GE Life Sciences, Buckinghamshire, UK) and both strands of the PCR products were sent for sequencing at STABVIDA (Lisbon, Portugal). Sequences obtained were edited and aligned using MEGA7 [59] to find a consensus sequence. These sequences were then compared with sequences from GenBank in BLAST searches, and species identification was obtained when at least 98% of similarity was found.

### 4.3. Pathogenicity Tests

Five representative isolates were selected from the fungal collection under study for pathogenicity tests, with the attention of selecting at least one isolate from the three different species of *Botryosphaeriaceae* identified three isolates of *Diplodia seriata* (Bt201, Bt204 and Bt212), one isolate of *Neofusicoccum parvum* (Bt216) and one isolate of *Diplodia mutila* (Bt218). To reproduce all the symptoms found in the field, three different pathogenicity tests were designed, being two performed on grapevines kept in a greenhouse and one on grapevines established in the field. Greenhouse pathogenicity tests were conducted on 1-year-old grafted Aragonez (=Tempranillo) plants, since is the most planted cultivar in Portugal and field tests were conducted on clusters of Cabernet Sauvignon which is currently the most planted cultivar worldwide.

#### 4.3.1. Pathogenicity tests on Tendrils and Leaves

Isolates were plated in Petri dishes containing 2% water agar with autoclaved pine needles (*Pinus pinea*) and incubated at 25 °C under fluorescent light, to promote sporulation [27,28,34]. Conidia were harvested from these plates by collecting the pycnidia formed on the pine needles into a 1.5 mL Eppendorf tube containing sterile distilled water and crushing them with the help of a pestle. These spore suspensions were filtered through cheesecloth and the concentrations were adjusted to 10^5^ spores/mL. To ensure full coverage of the tissues to be inoculated, 2 mL of each spore suspension was sprayed on the green tissues (leaves and tendrils) of 1-year-old grafted cuttings of cultivar Aragonez (=Tempranillo), individually potted in 1 L free draining bags containing a sandy soil mixture kept in a ventilated greenhouse at 24 °C under natural light. The aerial part of the plants was covered with a plastic bag for 3 days to promote infection, and the plants were assessed for the development of symptoms, namely necrosis on any of the inoculated organs or drying of leaves and tendrils, one week after inoculation. Ten plants were used for each isolate, while control plants were sprayed with sterile distilled water. The percentage of plants showing symptoms for each isolate was recorded.

#### 4.3.2. Pathogenicity Tests on Green Stems

One-year old grafted cuttings of cultivar Aragonez (=Tempranillo), individually potted in 1 L free draining bags containing a sandy soil mixture, kept in a ventilated greenhouse at 24 °C under natural light, were inoculated following the method described by Reis et al. [35]. Ten plants were used for each isolate, and the assessment for symptoms development, including external lesions or cankers, was performed one month after inoculation, by measuring the width and length of the lesions and calculating the elliptical area of the lesion. All statistical analysis was performed using the R program (www.r-project.org). Assumptions for variance analysis was assessed and when all the assumptions were not accomplished, the influence of distinct levels of one factor was assessed using the non-parametrical test of Kruskal-Wallis. In this case, when the significant differences were found (*p* < 0.05), the comparison between the distinct level was made using the ranks.

#### 4.3.3. Field Pathogenicity Tests on Clusters

Clusters of cultivar Cabernet Sauvignon on EL −25 to 27 stages, were selected from several field-grown grapevines in a vineyard located at ISA, Lisbon, Portugal. Artificial inoculations were performed with spore suspensions obtained as referred earlier for the pathogenicity tests on green tissues. Again, to ensure full coverage of the tissues to be inoculated, 1 ml of each spore suspension was sprayed on each cluster, which were covered individually with plastic bags for 3 days. Ten replicates were used for each isolate, and control cluster were sprayed with sterile distilled water. After one week, clusters were assessed for the development of symptoms such as dried or necrotic berries and inflorescences. These symptoms were quantified by using an adaptation of the EPPO protocol for evaluation of fungicides against *Botryotinia fuckeliana* on grapevine [60], where the percentage of the cluster area infected was assessed according to the following scale: 1 = no symptoms; 2 = 1–5%; 3 = 5–25%; 4 = 25–50%; 5 = >50%. The total percentage of clusters showing symptoms for each isolate was also recorded. The virulence of the different isolates was assessed through a multiple proportion test, comparing the proportion of symptomless samples of each modality towards the control. Boxplots for the infection rank of each isolate were defined to compare aggressiveness of the different isolates. All statistical analysis was performed using the R program (www.r-project.org).

In order to fulfil Koch’s postulates, samples were collected from all the different types of symptoms/lesions observed, and placed on PDA (Difco, USA, BD) amended with chloramphenicol (PanReac AppliChem, Darmstadt, Germany) at 250 mg/L to recovered the inoculated fungi.

## Figures and Tables

**Table 1:** *Botryosphaeriaceae* isolates obtained during the study, respective GenBank accession numbers, and percent identity when compared to reference sequences.

	Host		Origin		GenBank Accession Number		Percent Identity *	
Isolate Number	*(V. vinifera cvs.)*	Isolation Region		Species	ITS	Tef1-α	ITS	*Tef1*-α
Bt201	Seara Nova	Apex of the shoot	Vineyard 1	*Diplodia seriata*	MT786219	MW018672	100%	99%
Bt202	Alicante	Base of the shoot	Vineyard 2	*Diplodia seriata*	MT786220	MW018673	99%	99%
Bt203	Castelão	Rachis	Vineyard 3	*Diplodia seriata*	MT786221	MW018674	99%	100%
Bt204	Syrah	Base of the shoot	Vineyard 4	*Diplodia seriata*	MT786222	MW018675	100%	99%
Bt205	Castelão	Cluster	Vineyard 5	*Neofusicoccum parvum*	MT786223	MW018676	100%	99%
Bt206	Castelão	Leaf stem	Vineyard 5	*Diplodia seriata*	MT786224	MW018677	98%	99%
Bt207	Castelão	Base of the shoot	Vineyard 6	*Diplodia seriata*	MT786225	MW018678	100%	100%
Bt208	Aragonez	Apex of the shoot	Vineyard 7	*Diplodia seriata*	MT786226	MW018679	100%	99%
Bt209	Aragonez	Rachis	Vineyard 8	*Diplodia seriata*	MT786227	MW018680	100%	100%
Bt210	Castelão	Apex of the shoot	Vineyard 5	*Diplodia seriata*	MT786228	MW018681	99%	99%
Bt211	Arinto	Base of the shoot	Vineyard 9	*Neofusicoccum parvum*	MT786229	MW018682	99%	99%
Bt212	Castelão	Base of the shoot	Vineyard 10	*Diplodia seriata*	MT786230	MW018683	100%	100%
Bt213	Seara Nova	Apex of the shoot	Vineyard 1	*Diplodia seriata*	MT786231	MW018684	100%	99%
Bt214	Castelão	Apex of the shoot	Vineyard 11	*Diplodia seriata*	MT786232	MW018685	100%	100%
Bt215	Seara Nova	Rachis	Vineyard 12	*Diplodia seriata*	MT786233	MW018686	98%	100%
Bt216	Alicante	Apex of the shoot	Vineyard 13	*Neofusicoccum parvum*	MT786234	MW018687	100%	98%
Bt217	Aragonez	Apex of the shoot	Vineyard 14	*Neofusicoccum parvum*	MT786235	MW018688	100%	98%
Bt218	Aragonez	Base of the shoot	Vineyard 15	*Diplodia mutila*	MT786236	MW018689	100%	100%
Bt219	Alicante	Cluster	Vineyard 2	*Neofusicoccum parvum*	MT786237	MW018690	99%	100%
Bt220	Seara Nova	Base of the shoot	Vineyard 1	*Diplodia seriata*	MT786238	MW018691	99%	99%
Bt221	Seara Nova	Apex of the shoot	Vineyard 1	*Diplodia seriata*	MT786239	MW018692	100%	99%
Bt222	Castelão	Apex of the shoot	Vineyard 16	*Diplodia seriata*	MT786240	MW018693	100%	100%
Bt223	Alicante	Rachis	Vineyard 17	*Diplodia seriata*	MT786241	MW018694	99%	99%

* Reference sequences used—*D. seriata* ITS—AY259094, tef1-α—AY573220 [38]; *N. parvum* ITS—AY259098, tef1-α—AY573221 [38]; *D. mutila* ITS—KJ361837, tef1-α—KJ361829 [39].

**Table 2:** Conidial dimension of the *Botryosphaeriaceae* species under study.

Species/Isolate	Conidial dimensions	
	Length (μm)	Width (μm)
*D. seriata*		
Bt201	(21.27–) 25.22 ± 1.89 (–28.69)	(8.33–) 9.91 ± 0.88 (–11.75)
Bt202	*	
Bt203	(20.75–) 22.50 ± 1.12 (–24.23)	(6.71–) 8.17 ± 0.57 (–9.16)
Bt204	(20.43–) 22.65 ± 1.56 (–26.20)	(7.83–) 9.06 ± 0.76 (–10.93)
Bt206	(20.99–) 23.84 ± 1.52 (–26.36)	(7.71–) 9.23 ± 0.95 (–10.77)
Bt207	(19.61–) 23.58 ± 1.95 (–27.30)	(7.49–) 9.29 ± 0.81 (–11.26)
Bt208	(21.72–) 25.26 ± 1.41 (–28.73)	(9.53–) 11.13 ± 1.01 (–13.37)
Bt209	(19.61–) 22.17 ± 1.52 (24.53)	(7.20–) 9.07 ± 0.78 (–10.55)
Bt210		
Bt212	(21.27–) 23.91 ± 1.36 (–26.22)	(7.34–) 8.34 ± 0.60 (–9.36)
Bt213	(23.28–) 25.99 ± 1.58 (–29.18)	(7.73–) 9.03 ± 0.56 (–9.84)
Bt214		
Bt215	(21.02–) 24.58 ± 1.47 (–26.99)	(10.14–) 11.21 ± 0.66 (–12.83)
Bt220		
Bt221	(20.80–) 23.28 ± 1.35 (–25,53)	(8.59–) 9.99 ± 0.60 (–11.10)
Bt222		
Bt223	(21.42–) 22.94 ± 1.34 (–26.37)	(8.97–) 9.72 ± 0.66 (–11.40)
*N. parvum*		
Bt205	(15.51–) 17.18 ± 1.02 (–19.92)	(4.56–) 5.22 ± 0.43 (–6.11)
Bt211	(15,.2–) 18.28 ± 1.75 (–22,.4)	(4.73–) 5.33 ± 0.31 (–5.94)
Bt216	(11.39–) 13.71 ± 1.28 (–16.13)	(4.20–) 5.02 ± 0.45 (–6.02)
Bt217		
Bt219		
*D. mutila*	(20.80–) 23.50 ± 1.41 (–25.05)	(9.47–) 11.15 ± 1.57 (–16.25)
Bt218	(21.27–) 25.22 ± 1.89 (–28.69)	(8.33–) 9.91 ± 0.88 (–11.75)

* Isolate without sporulation.
